# Comparative analysis of two molecular tests for the detection of COVID-19 in Cameroon

**DOI:** 10.11604/pamj.2021.39.214.30718

**Published:** 2021-07-26

**Authors:** Guy Pascal Ngaba, Ginette Claude Mireille Kalla, Jules Clément Nguedia Assob, Abdel Jelil Njouendou, Christian Nelly Jembe, Emile Télesphore Mboudou, Francois-Xavier Mbopi-Keou

**Affiliations:** 1 Faculty of Medicine and Pharmaceutical Sciences, University of Douala, Douala, Cameroon,; 2 Gynaeco-Obstetrics and Pediatric Hospital Douala, Douala, Cameroon,; 3 Faculty of Medicine and Biomedical Sciences, University of Yaounde I, Yaounde, Cameroon,; 4 Faculty of Health Sciences, University of Buea, Buea, Cameroon,; 5 Ministry of Public Health, Deido Health District, Douala, Cameroon,; 6 The Institute for the Development of Africa (The-IDA), Yaounde, Cameroon

**Keywords:** Comparative analysis, AmpliQuick® SARS-CoV-2, Da An Gene RT-PCR, COVID-19, Douala, Cameroon

## Abstract

**Introduction:**

COVID-19 equation in Cameroon is yet to be resolved. There is an urgent need for a rapid response strategy to the increasing demand of polymerase chain reaction (PCR) test results for both patients, travelers and competitors to various games. We assessed the diagnostic performance of the AmpliQuick® SARS-CoV-2 against the classic Reverse transcription polymerase chain reaction (RT-PCR).

**Methods:**

a cross-sectional and comparative study was conducted from April 27^th^ to May 29^th^, 2021 in the city of Douala, Cameroon. The samples consisted of any nasopharyngeal sample received at the Douala Gynaeco-Obstetrics and Pediatric Hospital molecular biology laboratory, regardless of its origin. Sociodemographic parameters (age, profession (footballers, travelers, other), matrimonial status, nationality), comorbidity and known status of COVID-19, were recorded at collection sites. The main collection sites were the Deido Health District and the Douala Gynaeco-Obstetric and Pediatric Hospital. We performed testing using AmpliQuick® SARS-CoV-2 and the classic RT-PCR (Da An Gene Co.Ltd) on each sample during the one month period. Analytical performance parameters were determined. To determine the sensitivity of both methods, the Bayesian latent class model was performed on the median with 95% confidence interval, with p≤0.05 as significant level, as well as Kappa (κ) agreement between tests. An ethical clearance was sought and obtained from the University of Douala Institutional Ethics Committee.

**Results:**

a total of 1813 participants were enrolled, with the predominance of male (68.68%) and the age group 31 to 40 years old (31.33%). Most participants were married (53.46%) with only few with known COVID-19 status (5.47%). One thousand eight hundred and ten (1810) tests were performed by AMPLIQUICK® SARS-CoV-2 while only 1107 could be achieved with the classic RT-PCR. Over the study period, it was noted a drastic reduction in the time necessary to render results with the AMPLIQUICK® SARS-CoV-2 from 24 hours to 3 hours. The AMPLIQUICK® SARS-CoV-2 reduced technician hands-on time and its practicability was noticed based on the prefilled and ready-to-use microplates. A prevalence of 1.93% and 1.45% were obtained for AMPLIQUICK® SARS-CoV-2 and the classic RT-PCR respectively. This difference in the prevalence showed that AMPLIQUICK® SARS-CoV-2 (Sensitivity 83.5% [CI=64.6-95.2]) was more accurate than the classic RT-PCR (67.8% [CI=46.6-84.9]).

**Conclusion:**

it is time for a change of attitude to scale up the COVID-19 testing ability in Cameroon and the AMPLIQUICK® SARS-CoV-2 is an alternative diagnosis strategy which should help resolve the situation of timely and reliable results.

## Introduction

The ongoing COVID-19 pandemic has underpinned the central position of diagnostic testing in outbreak control worldwide [1,2] and especially in Cameroon where it is still a crucial equation to resolve [3]. It has revealed the importance of sequencing capacity for rapid pathogen and variant identification, and the benefit of open sharing of information between pathogen-specific experts and the global community. It has also, trigger the need to track pathogens evolution and spur rapid test development [2]. According to the World Health Organization (WHO), the gold standard to detect the coronavirus is the Real-time polymerase chain reaction (RT-PCR) using TaqMan probes, which precisely detect the presence of the virus [4]. Real-time PCR is hence recommended to detect SARS-CoV-2 infection if adequate response is to be ensured. Patently, PCR testing is highly suitable for large scale testing, as demonstrated daily by the millions of tests carried out to date. Since then, a large number of additional tests and improved protocols have been created by individual research groups and companies in many countries, targeting a range of SARS-CoV-2 specific sequences, some detecting multiple targets, others able to detect its presence in crude samples, and yet others generating results in minutes [4].

The acceleration of technology and expertise witnessed during the course of this pandemic is resulting in astonishing advances in convenience, speed and reach of PCR-based devices. More over ending the pandemic involves the accurate application of diagnostic testing in high volumes and the rapid use of the results to help implement the appropriate therapy and prevent further spread. However, PCR availability is restricted in most low and middle development countries (LMIC), like Cameroon. BIOSYNEX AmpliQuick® SARS-CoV-2 is an in vitro molecular diagnostic test for the qualitative detection of SARS-CoV-2 (virus responsible for COVID-19) from a ribonucleic acid (RNA) extract obtained from a single nasopharyngeal or oropharyngeal swab [5]. With its capacity to perform 96 samples in 90 minutes [5], there is hope that it can help in speeding up data analysis and timely release of results within a day. Among the currently use molecular diagnostic kits in the country is the RT-PCR (Da An Gene Co.Ltd) [6]. It specific primers and fluorescent probes are designed for the detection of 2019 Novel Coronavirus RNA in the specimens within 24 hours. Against this background, we assessed the diagnostic performance of the AmpliQuick® SARS-CoV-2 [5] against the classic RT-PCR [6] currently used in the country.

## Methods

**Study setting and design:** a cross-sectional and comparative study was conducted from April 27^th^ to May 29^th^, 2021 in the city of Douala, Cameroon. The main collection sites were the Deido Health District and the Douala Gynaeco-Obstetrics and Pediatric Hospital (HGOPED).

**Study population:** all individuals seeking COVID-19 tests received at the Deido Health District and at the HGOPED.

**Inclusion criteria:** all individuals of all age seeking COVID-19 tests received at the Deido Health District and at the HGOPED during the study period.

**Exclusion criteria:** critically ill patients.

**Data collection:** the samples consisted of nasopharyngeal swabs received at the molecular biology laboratory. Using a questionnaire, information on sociodemographic parameters (age, profession (footballers, travelers, other), matrimonial status, nationality), and known status of COVID-19, were recorded at collection sites from consented participants (and children and adolescent whose parent gave an assent). Each sample received was systematically analysed by DA AN GENE CO.LTD PCR (inactivation, manual extraction, amplification) [6] and by AMPLIQUICK® SARS-CoV-2 [5] and AMPLIQUICK® SARS-CoV-2 technique (was done without extraction using two kits namely a transport kit (AmpliQuick sample collection) and AmpliQuick viral lysis which replaces the classic step of prior the extraction) [5].

**Statistical analysis:** we assessed the most advantageous technique in terms of accuracy diagnostic performances. Statistical analysis of the data was performed using Microsoft Excel and IBM® SPSS version 20 software (Chicago, Illinois, USA). Analytical performance parameters (relating to how well the marker of interest is detected) were determined. To determine the sensitivity of both methods, the Bayesian latent class model was performed on the median with 95% confidence interval, with p≤0.05 as significant level, assuming no gold standard was performed as well as Kappa (κ) agreement between tests was performed [7,8].

**Ethical considerations:** an ethical clearance was sought and obtained from the University of Douala Institutional Ethics Committee (IEC) (2739IEC-UD/US2021M). This IEC is empowered by the Cameroon National Ethical Committee to assess all research on human health´s projects to be conducted in research and hospital institutions based in the Littoral Region. Each participant consented to be part of the study. Anonymity has been retained.

## Results

**Sociodemographic characteristics of the study population:**Table 1 presents the sociodemographic characteristics of the population. From a total of 1813 participants, the majority (71.59%) were Cameroonian, most of them in the age range 31 to 40 (31.33%). The male sex dominated (68.68%) and they were mostly married (53.46%). Only few participants (5%) knew their COVID-19 status.

**Table 1:** sociodemographic characteristics of the study population

Variables	Frequency	Percent
**Nationality**		
Cameroonian	1298	71.59
Other	515	28.41
Total	1813	
**Age range (years)**		
0-17	53	2.92
18-30	350	19.31
31-40	568	31.33
41-50	482	26.59
51-60	229	12.63
61-70	103	5.68
71+	28	1.54
Total	1813	
**Sex**		
Male	1226	68.68
Female	559	31.32
Total	1785	
**Marital status**		
Married	888	53.46
Single	743	44.73
Divorced	30	1.81
Total	1661	
**Known COVID-19 status**		
Yes	75	5.47
No	1295	94.53
Total	1370	
**Type of samples**		
Nasopharynx swab	1736	99.83
Saliva swab	2	0.12
Throat swab	1	0.06
Total	1739	

**Assessment of the diagnostic performances of PCR Da An Gene Co.Ltd and AmpliQuick:**Table 2 presents the diagnostic performances of PCR Da An Gene Co.Ltd/AmpliQuick tests performed during the same period. It appears that using the AmpliQuick molecular method the Laboratory technicians could analyze more samples (1810/1107) than with the PCR Da Angene procedure. Bayesian latent class model analysis assuming no gold standard was performed (Table 3). Bayesian latent class model assumed that all tests evaluated are imperfect. Values shown are estimated median with 95% confidence interval. The Ampliquick showed a higher sensitivity compared to the PCR Da An Gene Co.Ltd Method and a substantial agreement (Table 4).

**Table 2:** comparative performances of AmpliQuick and PCR Da An Gene Co. Ltd testing during the one-month screening period

Test	Number of tests performed	Number of positive	Prevalence (%)	95% CI
AmpliQuick	1810	35	1.93	[1.3-2.57]
PCR_DA AN GENE CO. LTD	1107	16	1.45	[0.74-2.15]

**Table 3:** bayesian latent class model analysis assuming no gold standard between AmpliQuick/PCR Da An Gene Co. Ltd methods

Parameters	Bayesian latent class model (%)
**PCR DA AN GENE CO. LTD**	
Sensitivity	67.8 (46.6 - 84.9)
Specificity	100 (100 - 100)
PPV	100 (100 - 100)
NPV	99.2 (98.3 - 99.6)
**Ampliquick**	
Sensitivity	83.5 (64.6 - 95.2)
Specificity	99.0 (98.1- 99.6)
PPV	68.3 (49.4 - 85.3)
NPV	99.6 (98.9 - 99.9)

PPV: positive predictive value; NPV: negative predictive value

**Table 4:** measure of agreement between tests Ampliquick vs PCR_DA AN GENE CO. LTD

Tests	Kappa (κ)	p-value	Interpretation
Ampliquick vs PCR_DA AN GENE CO. LTD	0.601	<0.001	substantial agreement

The κ statistic is interpreted as follows: 0 = agreement equivalent to chance; 0.10-0.20 = slight agreement; 0.21-0.40 = fair agreement; 0.41-0.60 = moderate agreement; 0.61-0.80 = substantial agreement; 0.81-0.99 = near-perfect agreement; and 1.00 = perfect agreement. Negative values indicate that the observed agreement is worse than what would be expected by chance

## Discussion

In Cameroon the demand for molecular testing is still to meet the satisfactory level. Only few laboratories countrywide are accredited to perform SARS-CoV-2 molecular testing. Results are usually release late (48 hours or more), leaving many people frustrated. From April 27^th^ to May 29^th^, 2021, a total of 1811 tests were performed by AMPLIQUICK® SARS-CoV-2 while only 1107 could be achieved with the classic RT-PCR. Over the study period, it was noted a drastic reduction in rendering results with the AMPLIQUICK® SARS-CoV-2 (4.7 times faster than the classic RT-PCR) from 24 hours to 3 hours. The AMPLIQUICK® SARS-CoV-2 reduced technician hands-on time and its practicability was noticed based on the prefilled and ready-to-use microplates. A prevalence of 1.93 % and 1.45% were obtained for AMPLIQUICK® SARS-CoV-2 and the classic RT-PCR respectively. This difference in the prevalence showed that AMPLIQUICK® SARS-CoV-2 (Sensitivity 83.5% [CI=64.6-95.2]) was more accurate than the classic RT-PCR (67.8% [CI=46.6-84.9]). The rapid implementation of COVID-19 tests requires critical assessment and adequate analyses of available tests. Proper laboratory diagnosis, techniques with high sensitivity and specificity are indispensable, considering that patients may have a low viral load when first infected. However, due to the intensive labor required by some techniques and the reagents involved, as well as the limited availability of kits, many diagnoses are based only on late-stage symptoms [4].

Although we have an increasing number of kits available using different reverse transcriptase, Taq polymerase and buffer combinations, all of which can detect SARS-CoV-2, the performance of different tests varies of course with regards to reproducibility and accuracy and how rapidly they return results but crucially, they should be sufficiently sensitive to detect the virus in pre-symptomatic individuals harboring a low viral load [9,10]. Since the spectrum of this disease in humans is not yet fully understood, countries have to optimize their ability to reach the maximum number of their citizens by selecting only appropriate kits for diagnosis having in mind the cost, the technician hands-on time, and performance. Low-cost diagnostic tests must be implemented for large-scale patients screening to confirm positive and/or negative cases of the new coronavirus in a context where social gathering are frequent for various reasons such as marriage, death and funeral celebrations. Given the many case scenario with possibility of viral transmission among people crowding to take part in several cultural and sports activity, there is an urgent need for a rapid response strategy to the increasing demand of PCR test results for both patients, travelers and players [11]. During the 2021 African Nation´s Championship, several cases of controversial results from the country´s medical experts marred the events image, something which the governing body wish to avoid given that similar competitions are going to take place in the country [12]. There needs to be global solidarity towards test access, and, importantly, infection control and diagnostic interventions need to be strongly intertwined to optimally combat current and future pandemics. Diagnostics should guide the choice of therapy and follow-up of therapy success timely [8,9].

**Limitations:** we were unable to record patients' satisfaction regarding the availability of COVID-19 results after sample collection.

**The Strengths:** a large sample size on participants from various origin. The two molecular analyses on samples were conducted in the same laboratory.

## Conclusion

We believe it is time for a change of attitude to scale up the COVID-19 testing ability in Cameroon and the AMPLIQUICK® SARS-CoV-2 is an alternative diagnosis strategy which should help resolve the situation of timely and reliable results.

### What is known about this topic

- The COVID-19 pandemic is yet to be resolved;
- The ongoing COVID-19 pandemic has underpinned the central position of diagnostic testing in outbreak control worldwide;
- Real-time PCR is hence recommended to detect SARS-CoV-2 infection if adequate response is to be ensured.

### What this study adds

- AmpliQuick® SARS-CoV-2 was more accurate than the classic RT-PCR;
- Over the study period, it was noted a drastic reduction in rendering results with the AmpliQuick® SARS-CoV-2 (4.7 times faster than the classic RT-PCR) from 24 hours to 3 hours;
- The AmpliQuick® SARS-CoV-2 reduced technician hands-on time and its practicability was noticed based on the prefilled and ready-to-use microplates.
